# IgE autoantibodies to nuclear antigens in patients with different connective tissue diseases: re-evaluation and novel findings

**DOI:** 10.3389/fimmu.2025.1483815

**Published:** 2025-03-20

**Authors:** Kathrin Kramer, Ann-Christin Pecher, Jörg Henes, Reinhild Klein

**Affiliations:** Department of Haematology, Oncology, Rheumatology, Immunology, University Hospital Tuebingen, Tuebingen, Germany

**Keywords:** IgE, antinuclear antibodies, systemic lupus erythematosus, Sjoegren’s syndrome, mixed connective tissue disease

## Abstract

**Introduction:**

Connective tissue diseases (CTD) are characterised by the overproduction of multiple autoantibodies, especially antinuclear antibodies (ANA) of the IgG type. Meanwhile, also IgE autoantibodies have been described. The aim was therefore, to establish an ELISA for the demonstration of IgE autoantibodies to SSA/Ro, SSB/La, RNP proteins and dsDNA in sera from patients with systemic lupus erythematosus (SLE), Sjoegren’s syndrome (SS), and mixed connective tissue disease (MCTD) to investigate their frequency and clinical relevance.

**Methods:**

Serum samples from 110 patients with SLE, 118 patients with SS, 41 patients with MCTD, and 73 controls were analysed by ELISA for IgE autoantibodies against dsDNA, SSA/Ro52, and SSA/Ro60, SSB/La, and RNP proteins using recombinant antigens. Patients were assessed for different clinical manifestations.

**Results:**

In SLE and SS, IgE anti-SSA/Ro52-, -SSA/Ro60- and -SSB/La-antibodies showed a significantly higher reactivity than in controls. IgE anti-dsDNA-antibodies were present in 66% of SLE patients. In SLE, there was a correlation of IgE anti-dsDNA- and -anti-SSA/Ro52-antibodies with disease activity and cutaneous manifestation. Neither IgE anti-SSA/Ro- nor -anti-SSB/La-antibodies were associated with distinct clinical manifestations in SS. Also, anti-RNP-antibodies were found to be of the IgE type (up to 90% in MCTD and 70% in SLE). In MCTD, IgE anti-Sm/RNPB- and -anti-RNP68-antibodies correlated with pulmonary manifestations. IgE anti-dsDNA- but not the other IgE autoantibodies decreased under immunosuppressive therapy.

**Conclusion:**

IgE anti-SSA/Ro-, -SSB/La-, -RNP-, and -dsDNA antibodies show a high frequency and specificity for the prevailing CTD. We confirmed an association of anti-dsDNA and anti-SSA/Ro52 antibodies with disease activity in SLE. In MCTD, there was an association of anti-Sm/RNP B and -RNP68 antibodies with pulmonary disorder.

## 1 Introduction

Connective tissue disease (CTD) is a collective term for systemic autoimmune disorders characterized by a large variety of clinical features and multisystemic involvement, which include systemic lupus erythematosus (SLE), Sjoegren’s Syndrome (SS), systemic sclerosis (SSc), mixed connective tissue disease (MCTD), and dermatomyositis/polymyositis (DM/PM) (1). CTDs present with various autoantibodies whose implications in the pathogenesis processes remain partly unclear, but there is evidence of direct involvement of some of these autoantibodies in tissue damage while some are just markers of disease development (2).

SLE is characterized by the production of autoantibodies against nuclear and cytoplasmatic antigens (2–4). This includes, among others, autoantibodies against DNA (single-stranded and double-stranded), histone, Sm, SSA/Ro, SSB/La, and RNP. The clinical symptoms vary from constitutional symptoms like fatigue, fever, and weight changes to organ symptoms of the musculoskeletal, renal, gastrointestinal, pulmonary, cardiovascular, neuropsychiatric, or cutaneous systems (3, 5, 6).

Patients with SS suffer predominantly from a ‘dry gland syndrome’ with sicca symptoms and florid salivary gland enlargement. Extraglandular features include fatigue, myalgia, neuropathies, nephropathies, interstitial pneumonitis and haematological abnormalities. The most relevant autoantibodies are anti-SSA/Ro and anti-SSB/La antibodies (2, 4, 7).

MCTD is characterized by an overlap of CTD symptoms, including features of SLE, myositis, rheumatoid arthritis, interstitial lung disease and SSc. It is defined by anti-U1 RNP antibodies (1, 8, 9).

In all CTDs, the diagnostically relevant antinuclear antibodies (ANA) are of the IgG type. Nevertheless, autoantibodies of IgE type were described in patients with SLE about five decades ago by Miyawaki et al. (10). Meanwhile, in several studies, ANA of type IgE reacting with different antigens, for example, dsDNA, Sm, RNP, SSA/Ro, SSB/La have been demonstrated in SLE-patients (11–15). An association of anti-dsDNA IgE antibodies with lupus nephritis has been discussed (12, 15, 16). In other CTD, ANA of type IgE have been rarely described (14, 17, 18). Recently, we showed that also the anti-topoisomerase I- as well as anticentromere antibodies (ACA) in SSc, belong quite frequently to the IgE class (56-77%), and a correlation of ACA with skin ulcers and the number of organ manifestations was observed (19).

Therefore, the present study aimed to re-evaluate the occurrence and clinical relevance of IgE autoantibodies against the most relevant antigens in patients with SLE, SS, and MCTD, namely towards SSA/Ro52, SSA/Ro60, SSB/La, U1-C RNP, RNP 68, RNP A, RNP B and dsDNA.

## 2 Patients

Serum samples from 110 patients with SLE, 118 patients with SS, and 41 patients with MCTD were analysed (**Supplementary Table S1A**). All patients were clinically and serologically well-defined, and the diagnosis followed the 2019 Classification Criteria for SLE (20), the 2017 Classification Criteria for Sjoegren’s syndrome (7), and the 1987 Classification Criteria for MCTD (21). Not all patients had sufficient serum quantities left to test for IgE autoantibodies to the various nuclear antigens; the number of serum samples evaluated is given in **Supplementary Table S1B**.

For examination of different organ manifestations, data collected included nail fold capillaroscopy (NC), left ventricular ejection fraction (LVEF in %) and systolic pulmonary arterial pressure (in mmHg) on echocardiography, resting and 24-hour Holter-electrocardiograms, cardiac magnetic resonance imaging (MRI), measurement of pulmonary arterial hypertension (PAH) on right heart catheterization (RHC), forced vital capacity (FVC) and diffusion capacity of the lungs for carbon monoxide (DLCO, % of predicted values), as well as high resolution computed tomography of the lungs. Pulmonary involvement was defined by the presence of PAH, interstitial lung fibrosis or alveolitis. N-terminal pro-brain natriuretic peptide (NT-proBNP), and troponin-I were determined for monitoring cardiac damage. Esophagogastroduodenoscopy was conducted to confirm gastrointestinal manifestations such as esophageal dysmotility, dyspepsia, esophageal and gastric ulcerations, pseudo-obstruction, bowel ischemia/perforation or enteritis. Myopathy was defined by evidence of proximal muscle weakness in the presence/absence of muscle enzyme elevations with at least one abnormal objective testing such as myopathic findings on electromyography (EMG) or inflammation, fibrosis, or necrosis on muscle biopsy. Differential blood count and liver enzymes were determined to assess haematological or hepatological diseases, and C-reactive protein (CRP) and C3/C4 complement were measured to evaluate inflammatory activity. The presence of nephrotic syndrome, persistent proteinuria of more than 500-1,000 mg/day with or without haematuria, or chronic kidney disease was the basis for the diagnosis of renal involvement. Kidney biopsy had been performed only in a few patients. In SLE patients, SLEDAI (Systemic Lupus Erythematosus Disease Activity Index) was additionally investigated (22, 23).

Treatment with steroids and/or immunosuppressive drugs (methotrexate, cyclophosphamide, azathioprine, mycophenolate mofetil, cyclosporine) was performed in 70% of the SLE-, 53% of the SS- and 76% of the MCTD patients (**Supplementary Table S1A**). Patients receiving monoclonal antibodies were excluded from the study.

To see whether the IgE antibodies to different nuclear antigens used in the present study may be non-specifically stimulated in patients with other chronic rheumatic diseases, sera from 25 patients with diffuse cutaneous SSc (dcSSc; 16 females, 9 males; age: median 46, range 21-77 years), and 25 patients with limited cutaneous SSc (lcSSc, formerly CREST-syndrome; Calcinosis-Raynaud phenomenon-Esophageal dysfunction-Telangiectasia; 24 females, 1 male; age: median 55, range 21-87 years), who had been already shown to have IgE antibodies to topoisomerase I and centromeric proteins (19) were included as ANA-positive controls; clinical data of these patients had been recently reported (19). Moreover, sera from 23 patients with fibromyalgia (FM; 16 females, 7 males; age: median 50, range 25-62 years) were analysed as an autoantibody negative control cohort. These sera were also used to calculate cut-off values (see below).

All patients were seen by one of the authors (ACP, JH) in the Rheumatological Outpatient Clinic of the Department of Internal Medicine, University Hospital in Tübingen, and all of them were diagnosed according to current international classification criteria (7, 20, 21). The study was approved by the local ethics commission (No. 076/2012BO1; 647/2016BO2) and performed according to the Helsinki guidelines. All patients provided written informed consent.

## 3 Materials and methods

### 3.1 Enzyme-linked immunosorbent assay

IgG antibodies to SSA/Ro52 (52 kDa), SSA/Ro60 (60 kDa), SSB/La, Sm/RNP B (U1-snRNP B/B’), U1-C RNP, RNP 68 (U1-snRNP 68/70 kDa), and RNP A (U1-snRNP A) [all obtained from Diarect AG, Freiburg (Germany)] were detected by an in-house ELISA as described for IgG antibodies to topoisomerase-I earlier (24). For the assessment of IgE antibodies, the recently described assay for the demonstration of IgE antibodies to centromeres and topoisomerase was adapted (19). Optimal antigen and serum dilutions were determined by serial dilutions before the study (data not shown). 96-well microtiter plates (Nunc™ Maxisorp, Thermo Fisher Scientific, Roskilde, Denmark) were coated with SSA/Ro52, SSA/Ro60, SSB/La, Sm/RNP B, U1-C RNP, RNP 68, and RNP A in a concentration of 0.5µg/ml for the detection of IgE- and of 0.33µg/ml for the detection of IgG antibodies. Patients’ sera were diluted 1:2 for the analysis of IgE- and 1:5,000 for the analysis of IgG antibodies. Bound antibodies were detected using peroxidase-conjugated AffiniPure goat anti-human IgG (Jackson ImmunoResearch, West Grove, USA), and goat anti-human IgE (ϵ-chain specific)-antibodies (Sigma-Aldrich, München, Germany) diluted 1:3,000 and 1:500, respectively. A serum with a high and a serum with a moderate autoantibody reactivity plus an antibody-negative serum were used as controls in each test. These samples were tested on each plate to normalise the results and minimise discrepancies between plates.

All tests were performed in duplicates. As a substrate, o-phenylenediamine was used. Bound peroxidase-conjugated antibodies led to a change of colour, which was measured photometrically in an ELISA reader as optical density (Absorbance microplate reader SLT Rainbow, TECAN Group AG, Männedorf, Switzerland). Analysis was performed with the Magellan™ Data Analysis Software (TECAN Group AG, Männedorf, Switzerland).

Optical density multiplied by 1,000 (ODx1,000) was used as an indicator of antibody reactivity in the ELISA.

Anti-dsDNA-IgG antibodies were detected using a commercial kit (kindly provided by diagnostic-a, Ebringen, Germany). To detect dsDNA-IgE autoantibodies, the Goat Anti-Human IgE (ϵ-chain specific)-antibodies (Sigma-Aldrich, München, Germany) were used instead of the anti-human IgG antibody provided with the ELISA kit. The reactivity of IgG anti-dsDNA-antibodies is given as IU/ml according to the manufacturer’s instructions, and the reactivity of IgE anti-dsDNA-antibodies is reported as ODx1,000.

The normal ranges for the different autoantibodies were determined with sera from the antibody negative FM patients. Thus, the cut off values were calculated using the mean of the IgE reactivities with the different antigens plus three times the standard deviation. In this way, values ranging from 44 to 53 ODx1,000 were determined for the IgE antibodies against the various antigens. To simplify the representations, we chose a cut off value of 50 ODx1,000 for all antibodies. ROC curves were used to check that with this threshold the specificity for all antibodies was over 95% (data not shown).

### 3.2 Statistics

GraphPad Prism Version 9.2.0 was used for statistical analyses. The normal distribution of the values in the distinct groups was evaluated with the Shapiro-Wilk-Normality test. Merely in the FM-patients, some parameters were normally distributed; we, therefore, applied only non-parametric tests. For the analysis of unpaired reactivity between two groups, the Mann-Whitney-U-Test was used. For multiple comparisons, Kruskal-Wallis followed by Dunn’s test was applied. Chi-Squared-Test was used to analyse nominal attributes of organ manifestations and antibodies without normal distribution. P-values <0.05 were considered statistically significant.

The correlation of non-parametric data of IgG- and IgE antibodies was calculated using Spearman’s correlation coefficient. R < 0.5 was considered weak, between 0.5 and 0.7 moderate, and > 0.7 high correlation (25).

## 4 Results

### 4.1 Demonstration of IgE antibodies to dsDNA, SSA/Ro52, SSA/Ro60, SSB/La, U1-C RNP, RNP 68, RNP A, and Sm/RNP B in patients with different CTD

**Figure 1:**
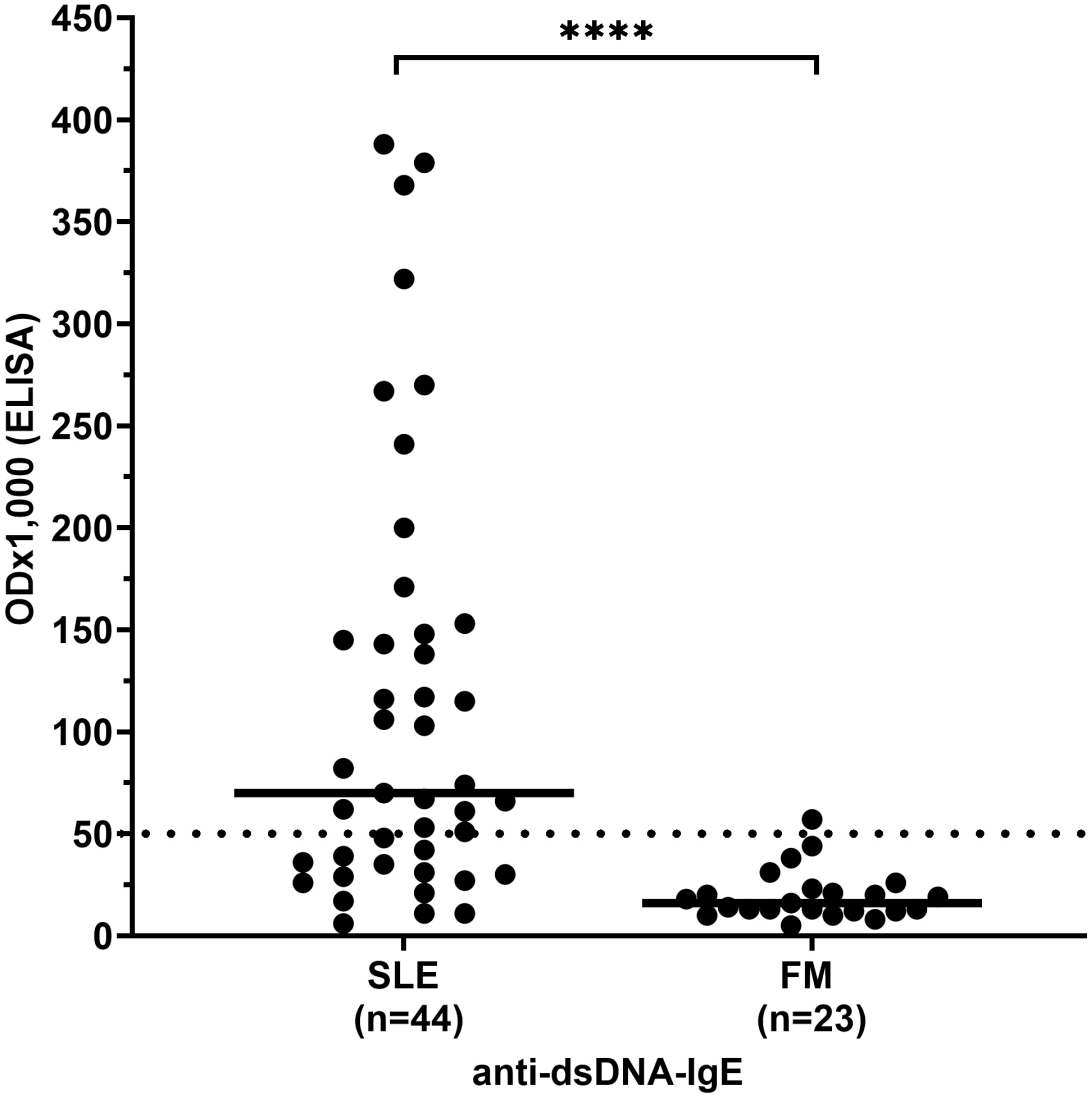
IgE antibody reactivity to dsDNA in patients with systemic lupus erythematosus (SLE) compared to controls. FM, fibromyalgia syndrome; OD, optical density. Individual values are given. - = median; — =threshold value; ****p<0,0001 (Mann-Whitney-U-test).

Antibody reactivity, i.e. ODx1,000 as defined in ‘methods’, and frequency of anti-dsDNA antibodies of the IgE type were significantly higher in SLE patients (median 75 and 64%, respectively) than in FM patients (10 and 4%, respectively, p< 0.0001; **Figure 1**).

**Figure 2:**
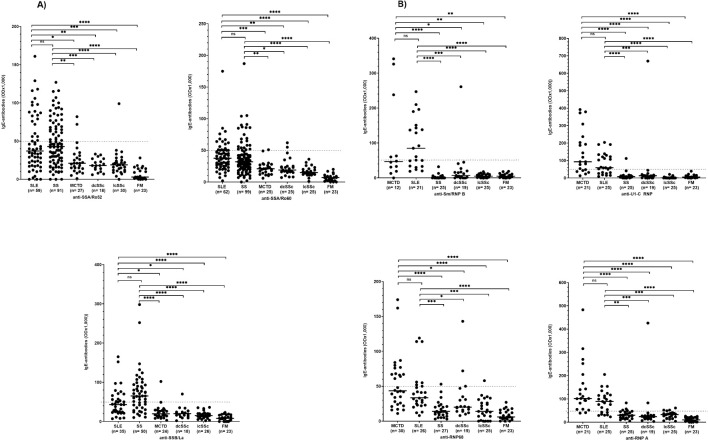
IgE antibody reactivity to SSA/Ro52, SSA/Ro60, and SSB/La **(A)** as well as to RNP-proteins **(B)** in patients with different CTD. SLE, systemic lupus erythematosus; dcSSc, diffuse cutaneous systemic sclerosis; lcSSc, limited cutaneous systemic sclerosis; MCTD, mixed connective tissue disease; FM, fibromyalgia syndrome; OD, optical density. ns=not significant. Individual values are given; -, median; —, threshold value; *p=<0,05; **p=<0,01; ***p=<0,001; ****p<0,0001 (Kruskal-Wallis followed by Dunn’s test).

The reactivity of anti-SSA/Ro52-, -SSA/Ro60-, and -SSB/La-antibodies of the IgE type was significantly higher in patients with SS and SLE than in patients with other CTD (**Figure 2A**). There was no significant difference in the reactivity of these antibodies between patients with SS and SLE (**Figure 2A**).

Also, the frequency of anti-SSA/Ro52-, and -SSA/Ro68-IgE antibodies was similar in patients with SLE and SS. In both diseases it was significantly higher than in patients with other CTD (**Table 1A**). The frequency of anti-SSB/La-IgE antibodies was significantly higher in patients with SS (68%) compared to SLE (40%; p < 0.05), and significantly higher in both diseases than in other CTD (**Table 1A**).

**Table 1A:** Frequency of IgE anti-SSA/Ro52-, -SSA/Ro60-, and -SSB/La-antibodies in patients with different connective tissue diseases and controls.

Diseases	Number of patients antibody positive/number tested (% positive)		
	Anti-SSA/Ro 52	Anti-SSA/Ro60	Anti-SSB/La
SLE	21/59 (36)	19/62 (31)	14/35 (40)
SS	35/91 (39)	23/99 (23)	34/50 (68)^*)^
MCTD	2/27 (7) ^****)^	1/25 (4) ^****)^	1/24 (4) ^****)^
dcSSc	0/18 ^****)^	2/25 (8) ^****)^	1/19 (6) ^****)^
lcSSc	1/30 (3) ^****)^	0/25 ^****)^	0/26 (0) ^****)^
FM	0/23 ^****)^	0/23 ^****)^	0/23 ^****)^

^*)^ p < 0.05 as compared to patients with SLE.

^****)^ p < 0.0001 as compared to patients with SLE and SS.

SLE, systemic lupus erythematosus; SS, Sjoegren syndrome; MCTD, mixed connective tissue disease; dcSSc, diffuse cutaneous systemic sclerosis; lcSSc, limited cutaneous systemic sclerosis; FM, fibromyalgia syndrome.

The reactivity of anti-Sm/RNP B, -U1-C RNP-, -RNP 68-, and -RNP A-antibodies of the IgE type did not differ between patients with SLE and MCTD but was significantly higher in these patients than in patients with other CTD (**Figure 2B**).

Analysing the frequency of anti-Sm/RNP B antibodies of the IgE type, 67% of the SLE and 42% of MCTD patients were positive, while the antibodies were barely detectable in patients with other CTD (**Table 1B**). Also, anti-U1-C RNP-, -RNP 68- and -RNP A-antibodies were found in a significantly higher frequency in patients with MCTD and SLE compared to the other collagen disorders.

**Table 1B:** Frequency of IgE anti-U1-C RNP-/-RNP68-/-RNP A-/-RNPA B (Sm)- antibodies in patients with different connective tissue diseases and controls.

Diseases	Number of patients antibody positive/number tested (% positive)			
	Anti-Sm/RNP B	Anti-U1-C RNP	Anti-RNP 68	Anti-RNP A
MCTD	5/12 (42)	15/21 (71)	13/30 (43)	19/21 (91)
SLE	14/21 (67)	17/25 (68)	7/26 (27)	18/25 (72)
SS	0/25 ^****)^	1/25 (4) ^****)^	1/27 (4) ^****)^	4/25 (16) ^****)^
dcSSc	1/19 (5) ^**)^	1/19 (5) ^****)^	3/19 (16) ^**)^	2/19 (11) ^****)^
lcSSc	0/25 ^****)^	0/25 ^****)^	1/25 (4) ^****)^	3/25 (12) ^****)^
FM	0/23 ^****)^	0/23 ^****)^	0/25 ^****)^	0/23 ^****)^

^*)^ p < 0.05 ^**)^ p < 0.01 ^****)^ p < 0.0001 as compared to patients with MCTD.

SLE, systemic lupus erythematosus; S, Sjogren syndrome; MCT, mixed connective tissue disease; dcSSc, diffuse cutaneous systemic sclerosis; lcSSc, limited cutaneous systemic sclerosis; FM, fibromyalgia syndrome.

### 4.2 Correlation between IgE- and IgG reactivity of antibodies to different nuclear antigens

All serum samples were also analysed for IgG antibodies to the respective antigens (**Supplementary Table S1A**). It became evident, that antibodies to dsDNA, SSA/Ro58, SSA/Ro62, SSB/La, and RNP 68 of the IgE type were in most instances associated with IgG antibodies independently from the kind of target antigen or diagnosis and hardly found to be exclusive of the IgE type. In contrast, IgE anti-U1-C RNP- and -RNP A-antibodies without the corresponding IgG antibodies were observed in 29% and 20%, respectively of SLE patients. In MCTD, 17% had only anti-Sm/RNP B-, 15% only anti-U1-C RNP-, and 16% only anti-RNP A antibodies without the corresponding IgG antibodies (**Supplementary Table S2A–H**).

The correlation between the reactivity of IgE- and IgG antibodies was moderate for all antibodies (r between 0.56 and 0.77) (**Supplementary Figure S1A–H**).

### 4.3 Influence of immunosuppressive therapy on IgE antibody reactivity towards nuclear antigens

**Figure 3:**
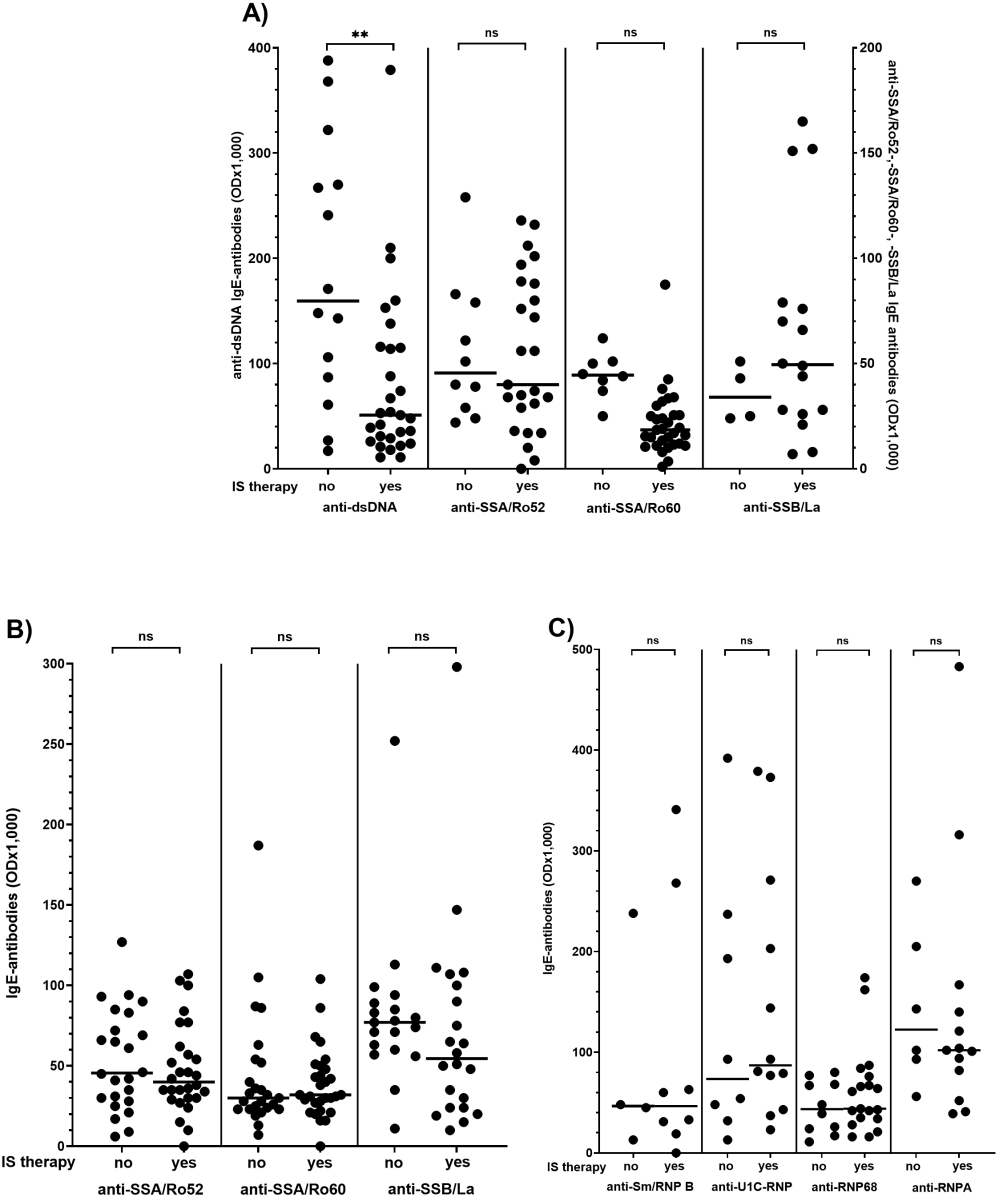
Effect of immunosuppressive (IS) therapy on IgE antibody reactivity in patients with systemic lupus erythematosus **(A)**, Sjoegren’s syndrome **(B)** and mixed connective tissue disease **(C)** Individual values are given. - = median; — = threshold value. ns, not significant; OD, optical density; **p<0,01 (Mann-Whitney-U-test).

As shown in **Figure 3**, the reactivity of anti-dsDNA antibodies of the IgE type was significantly lower in SLE patients receiving immunosuppressive therapy than in patients without treatment (**Figure 3A**). IgE antibodies to other nuclear antigens were not affected by immunosuppressive therapy in any of the other CTDs (**Figure 3B, C**).

### 4.4 Association of IgE autoantibodies in different connective tissue disorders with clinical manifestations and disease activity

#### 4.4.1 SLE

**Figure 4:**
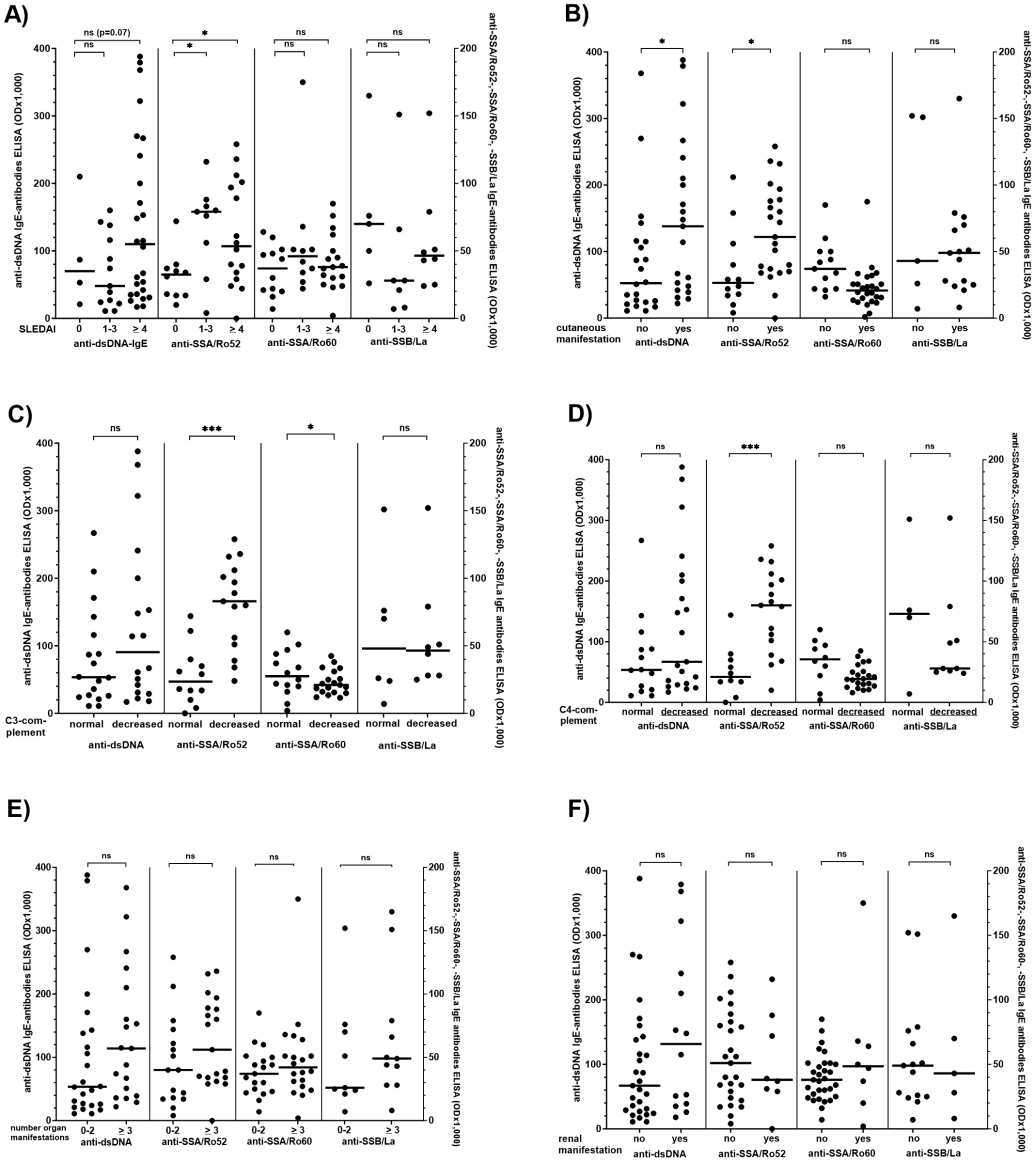
Comparison of the IgE anti-dsDNA-, -SSA/Ro52-, -SSA/Ro60-, and -SSB/La-antibody reactivity with disease activity, extent of different organ manifestations, and complement levels in patients with SLE. **(A)** Systemic Lupus Erythematosus Disease Activity Index (SLEDAI), **(B)** number of cutaneous manifestations, **(C)** C3 complement concentration, **(D)** C4 complement concentration, **(E)** number of organ manifestations, **(F)** renal manifestation (defined by the presence of nephrotic syndrome, persistent proteinuria of more than 500-1,000 mg/day with or without haematuria, or chronic kidney disease In only 7 of the 14 patients with renal manifestation kidney biopsy had been performed. There was no association between the presence of IgE ds DNA autoantibodies and lupus nephritis class I/II and III/IV). Individual values are given. - = median; — = threshold value. ns, not significant; OD, optical density. *p<0,05; ***p<0,01. Mann-Whitney-U-test was performed to compare two groups, Kruskal-Wallis followed by Dunn’s test when more than two groups were analysed.

There are several reports on the association of anti-dsDNA IgE antibody reactivity and disease activity in patients with SLE, and our first step was to confirm their reproducibility (26, 27). Therefore, reactivity and frequency of IgE type antibodies against dsDNA, SSA/Ro52, SSA/Ro60, and SSB/La were analysed concerning clinical manifestations including SLEDAI, number of organ manifestations, cutaneous, renal, pulmonary, cardiac, musculoskeletal, gastrointestinal, and haematological manifestation in 90 patients with SLE (**Figure 4**, **Supplementary Table S3**).

Reactivity and frequency of IgE anti-SSA/Ro52-antibodies were significantly increased in patients with a SLEDAI >1 as compared to a SLEDAI of 0 (**Figure 4A**, **Supplementary Table S3**); for IgE dsDNA antibodies there was only a trend comparing patients with a SLEDAI of 0 and those with a SLEDAI of > 4 (p=0.07). Correlation between the SLEDAI and IgE anti-SSA/Ro52 or IgE anti-dsDNA antibody reactivity in all patients was only weak (r=0.30 and 0.29, respectively).

Moreover, patients with cutaneous manifestations had significantly higher IgE anti-dsDNA- and -SSA/Ro52-antibody reactivity as compared to patients without cutaneous manifestation (**Figure 4B**). Also, anti-SSA/Ro52- and – to a lesser extent - anti-SSA/Ro60-IgE reactivity was significantly higher in patients with low as compared to patients with normal serum C3- or C4-complement levels (**Figure 4C, D**), and there was a moderate inverse correlation between C3-complement levels and anti-SSA/Ro60-IgE reactivity (r=-0.57; p < 0.05). Other IgE antibodies were correlated neither with C3- nor C4-complement levels.

We did not observe an association of IgE-antibodies with the number of organs involved (**Figure 4E**, **Supplementary Table S3**). For the assessment of an association between the antibodies and kidney involvement, the number of patients in whom kidney biopsy had been performed, (n=7) was too low to draw any reliable conclusions. Four of these seven patients suffered from class I/II nephritis, and two were positive for IgE anti-dsDNA antibodies. However, there was a moderate correlation between serum creatinine and IgE anti-SSA/Ro52 antibodies (r=0,66, p < 0.01), which was not observed for any of the other autoantibodies including IgE anti-dsDNA-antibodies (**Figure 4F**, **Supplementary Table S3**).

Since anti-dsDNA IgE reactivity decreased under IS therapy, we compared the clinical parameters and IgE dsDNA reactivity only in the untreated patients. In this distinct group, the SLEDAI significantly correlated with the antibody reactivity (r=0.70; p<0.01).

#### 4.4.2 SS

Analysing 55 patients with SS, there was no association between the reactivity of anti-SSA/Ro52-/-SSA/Ro60- and -SSB/La-IgE antibodies and the number of organ manifestations, presence of sicca-syndrome, musculoskeletal, or cutaneous symptoms (**Table 2A**). Also, antibody frequency was not different between patients with or without distinct clinical symptoms or pathological laboratory parameters (**Supplementary Table S4**).

**Table 2A:** IgE anti-SSA/Ro52, -SSA/Ro60, and -SSB/La reactivity in different organ manifestations in patients with Sjoegren’s Syndrome.

	Number organ manifestations		Presence of					
			Sicca-syndrome		Musculoskeletal manifestations		Cutaneous manifestations	
	0-2	>3	no	yes	no	yes	no	yes
Anti-SSA/Ro52-IgE								
Number patients	38	14	7	45	35	17	41	11
Mean ± std^*)^	51.0 ± 29.0	49.0 ± 29.0	50.3 ± 49.3	50.2 ± 25.6	47.7 ± 29.3	55.4 ± 29.1	52.4 ± 29.8	42.0 ± 26.3
median^*)^	43.0	41.0	34.0	44.0	41.0	46.0	46.0	35.0
range^*)^	0-127	15-107	0-127	9-103	0-127	15-107	0-127	21-86
Anti-SSA/Ro60-IgE								
Number patients	39	15	7	47	35	19	42	12
Mean ± std^*)^	42.0 ± 33.0	40.0 ± 25.0	34.4 ± 32.6	42.1 ± 30.3	38.1 ± 22.9	46.6 ± 41.0	41.1 ± 30.5	41.1 ± 31.3
median^*)^	32.0	31.0	30.0	32.0	32.0	31.0	31.0	32.0
range^*)^	0-187	16-104	0-104	7-187	0-105	16-187	0-187	7-105
Anti-SSB/La-IgE								
Number patients	29	12	4	37	24	17	34	7
Mean ± std^*)^	82,0 ± 61,0	61,0 ± 39,0	75,0 ± 47,0	75,6 ± 57,4	74,1 ± 56,9	77,6 ± 56,4	81,8 ± 58,6	45,3 ± 27,0
median^*)^	74,0	61,0	89,5	65,0	75,5	64,0	74,5	35,0
range^*)^	10-298	15-147	10-111	11-298	10-298	19-252	10-298	15-90

^*)^ given as OD (optical density) x 1,000.

#### 4.4.3 MCTD

The reactivity and frequency of IgE antibodies of the RNP group were analysed for their association with different clinical manifestations in 38 MCTD patients. There was a statistically significant difference in reactivity and frequency of anti-Sm/RNP B- and -RNP68-antibodies comparing patients with or without pulmonary manifestations (p<0.05) (**Table 2B**, **Supplementary Table S5**). There was no association of these antibodies with other clinical parameters. Also, other IgE anti-RNP-antibodies were not associated with distinct clinical manifestations.

**Table 2B:** IgE anti-Sm/RNP B-, -U1C-RNP-, -RNP68- und -RNP A reactivity in different organ manifestations in patients with MCTD.

	Number organ manifestations		Presence of					
			Cutaneous manifestations		Musculoskeletal manifestations		Pulmonary manifestations	
	0-2	>3	no	yes	no	yes	no	yes
Anti-Sm/RNP B								
Number patients	3	9	3	9	4	8	9	**3**
Mean ± std^*)^	110.3 ± 110.6	92.0 ± 123.5	120.3 ± 127.9	88.7 ± 118.5	91.0 ± 98.2	99.4 ± 129.9	54.4 ± 71.5	223.0 ± 145.8^**)^
median^*)^	48.0	33.0	48.0	33.0	46.5	45.5	33.0	268.0^**)^
**range** ^*)^	45-238	0-341	45-268	0-341	33-238	0-341	0-238	**60-341**
Anti-U1C-RNP								
Number patients	5	15	7	13	4	16	14	6
Mean ± std^*)^	157.8 ± 147.7	138.4 ± 123.1	163.7 ± 127.2	132.2 ± 128.9	85.0 ± 101.9	157.8 ± 129.9	123.4 ± 128.7	189.5 ± 116.0
median^*)^	93.0	81.0	144.0	81.0	40.0	93.0	67.5	168.5
**range** ^*)^	32-379	13-392	32-379	45-268	23-237	13-392	13-392	77-373
Anti-RNP68								
Number patients	11	18	12	17	9	20	21	**8**
Mean ± std^*)^	41.6 ± 19.2	64.6 ± 44.9	52.8 ± 19.4	57.9 ± 48.2	42.3 ± 24.4	61.9 ± 42.6	48.8 ± 36.6	74.3 ± 39.8^**)^
median^*)^	39.039	62.5	46.0	42.0	39.0	52.5	42	71.0^**)^
range^*)^	11-68	16-174	26-87	11-174	11-80	16-174	11-174	**39-162**
Anti-RNPA								
Number patients	5	14	8	11	5	14	12	7
Mean ± std^*)^	129.8 ± 86.4	147.3 ± 120.2	153.0 ± 74.8	135.2 ± 133.5	129.4 ± 86.89	147.4 ± 120.1	107.3 ± 65.0	203.3 ± 148.1
median^*)^	102.0	103.0	130.5	94.0	102.0	103.0	101.5	140.0
range^*)^	41-270	39-483	93-316	39-483	39-270	41-483	39-270	82-483

^*)^ given as OD (optical density) x 1,000.

^**)^ significant as compared to patients without pulmonary manifestation p < 0.05.

Bold text indicates significant values.

Four SLE- and three MCTD patients had only IgE- but no IgG anti-U1C RNP-antibodies. Moreover, anti-RNP A antibodies and anti-Sm/RNP B antibodies were only of the IgE type in three and two MCTD patients, respectively (**Supplementary Table S2**). Analysing these patients’ disease activity, it became evident, that it was strongly increased in all of them as indicated by high SLEDAI, low complement levels and increased number of organs involved; however, the number of patients is too low to perform statistical analysis.

## 5 Discussion

In the present study, we showed that all antibodies to nuclear antigens in patients with SLE, SS, and MCTD belong not only to the IgG- but also to the IgE type, although to varying extents. For instance, in SLE, IgE anti-dsDNA antibodies were present in 66% and anti-SSA/Ro or anti-SSB/La antibodies in 30-40% of the patients. In patients with SS, even 68% had anti-SSB/La IgE antibodies. As expected, these antibodies were rarely found in patients suffering from MCTD, dcSSc, or lcSSc, underlining their disease-specificity. In MCTD, IgE autoantibodies to U1 C-RNP and RNP A predominated (91 and 71%, respectively), but also anti-Sm/RNP B- and -RNP A-IgE autoantibodies exist.

For the detection of IgE autoantibodies to nuclear antigens, we have developed an in-house ELISA using recombinant antigens in their native conformation. IgE antibody reactivity was much lower than IgG reactivity, which may be due to lower serum IgE levels in general or a lower affinity or avidity of the IgE antibodies to the respective antigens as compared to IgG antibodies. The serum concentration in the assays had to be, therefore, increased to demonstrate IgE autoantibodies. Despite this, the established IgE assays proved specific as shown by analysing adequate negative controls. In most instances, the IgE antibodies were associated with IgG antibodies to the respective antigens, and for all of them, there was a moderate correlation between IgE- and IgG reactivity. Nevertheless, in some patients, antibodies to the RNP-group were exclusively of the IgE type, and these patients seemed to suffer from a more active disease.

Besides their existence, we also analysed the clinical relevance of the different IgE-autoantibodies. In accordance with Dema et al. (12), in the present study, we found only a trend towards higher anti-dsDNA-IgE antibodies in SLE patients with increased SLEDAI. In contrast, IgE anti-SSA/Ro52- (but not IgE anti-SSA/Ro60-) antibody reactivity was significantly higher in patients with a SLEDAI >1 than in patients with inactive SLE. Also, C3-/C4-complement levels as a marker for disease activity (28, 29) were significantly lower in patients with increased IgE anti-SSA/Ro52-antibody reactivity. Moreover, there was a moderate correlation between serum creatinine levels indicating renal involvement and IgE anti-SSA/Ro52-antibody reactivity. Unfortunately, kidney biopsy had been performed only in a minority of our SLE patients so we cannot state their association with nephritis. However, we found an association between skin manifestations in SLE patients and elevated IgE anti-dsDNA- and -anti-SSA/Ro52 antibodies, an observation that had not yet been reported by other authors. In this respect, it is noteworthy that IgE generally binds with high affinity to the Fcε receptor I which is expressed by mast cells, basophils, dendritic cells, and Langerhans cells. Langerhans cells seem to be involved in photosensitivity in SLE (30), and one could, therefore, speculate, that this might be linked to the well-known clinical observation that cutaneous manifestations – besides others - frequently develop after sun exposure (31).

For the IgE antibodies to ribonucleoproteins RNP 68 and Sm/RNP B, we found an association with pulmonary manifestations in MCTD but no other clinical manifestations. Indeed, there is evidence that T helper type 2 (Th2) reactivity, which mediates the production of IgE antibodies, promotes pulmonary fibrosis in an MCTD-like mouse model. Thus, in this model it was shown that immunization with RNP 68 (U1-snRNP) led not only to the induction of anti-RNP 68/U1-snRNP antibodies but also the development of MCTD-like lung disease (14). IgE deficiency completely prevents lung disease (14) as also shown in the development of nephritis in SLE (15, 26, 32). In SLE we observed no association of IgE anti-RNP-antibodies with any organ manifestation.

In a further step, we wanted to see whether the IgE-autoantibodies are influenced by immunosuppressive therapy. It became evident, that only the reactivity of IgE-anti-dsDNA antibodies is influences by this kind of treatment. None of the other IgE autoantibody specificities was affected. This parallels the clinically well-known effect of immunosuppressive therapy on IgG anti-dsDNA antibodies but no other IgG-ANA-specificities and argues for their relevance in pathogenesis and disease activity. Nevertheless, it is surprising and incomprehensible, that the IgE anti-SSA/Ro52-antibodies, which were also associated with some clinical parameters, were not influenced.

Primarily, IgE immunoglobulins have been linked mainly to allergic and parasitic disorders. Thus, binding of IgE to the FcεRI on mast cells and basophils leads to their activation and degranulation, resulting in the release and biosynthesis of proinflammatory mediators (15, 33), that trigger different allergic reactions or even an anaphylactic shock. However, there is no doubt that IgE *auto*antibodies also exist and may play a role in the pathogenesis of autoimmune disorders (15, 34). These antibodies can activate Fcε receptors without inducing degranulation but resulting in the activation of plasmacytoid dendritic cells hereby upregulating the secretion of type I-interferons, the promotion of basophils into lymph nodes, the activation not only of T helper type 2 cells but also CD8+ T-cells and the induction of plasma cell differentiation (13, 26, 27, 35–37). Although the specific role of the antinuclear antibodies of the IgE type in the distinct clinical entities remains still obscure, their existence, however, opened new therapeutic options. IgE deficiency in mice prevented or diminished autoimmune reactions. Therefore, a therapeutic strategy to eliminate IgE antibodies in humans with autoimmune diseases, as already performed for allergies, is reasonable. Monoclonal anti-IgE antibodies have been evaluated in patients with CTD, especially SLE. Indeed, in a preliminary study a decrease of the SLEDAI, shown in the present study to correlate with IgE anti-SSA/Ro antibodies, was observed during treatment with Omalizumab, which is a humanised monoclonal anti-IgE antibody that targets free IgE and prevents its binding to Fcε receptors. Therefore, the determination of IgE autoantibodies in CTD could become relevant for therapeutic purposes in the future.

Our study has several limitations. The number of patients in some clinical manifestations was rather small, which affected the statistical power of the analysis. Moreover, the IgE autoantibodies have been analysed retrospectively, and due to limited amounts of serum samples and antigens, we could not test all sera for all IgE antibody specificities. A prospective longitudinal study of the antibodies would be important to understand how they might be influenced by disease relapses, environmental factors, or therapeutic strategies. Additionally, the importance of determining cut-off values should be considered when reporting the incidence of autoantibodies in clinical settings. In our study, we calculated the mean reactivity (ODx1,000) values plus three times the standard deviation determined by autoantibody-negative individuals. The reliability of this procedure was confirmed by calculating receiver operating curves (data not shown). It is also important to note that assays using different antigens (native vs non-native, recombinant vs purified) can significantly influence the results, which may explain the discrepancies in frequencies and reactivities of IgE antibodies in distinct clinical entities reported by different research groups.

In conclusion, our data confirm and validate previous observations, indicating that all ANA in CTD may be of the IgE type (12, 14, 18, 27) and that at least some of them may correlate with disease activity or organ involvement. However, we could also extend the published data. Thus, we showed that in MCTD not only do the anti-U1 C-RNP- and -RNP 68 (U1 snRNP68/70) antibodies belong quite frequently to the IgE-type – as already reported -, but also the anti-Sm/RNP B- and -RNP A- autoantibodies. Interestingly, especially the anti-Sm/RNP B- and -RNP 68-antibodies were significantly associated with pulmonary manifestations in the MCTD patients. In contrast, in patients with SS, we found no association between IgE-anti-SSA- or -SSB-antibodies and clinical activity, a fact which seems logical since this is already known for the IgG-antibodies but had not yet been analysed in detail for the IgE-antibodies. In contrast, in SLE, the anti-SSA antibodies correlated with increased disease activity as defined by higher SLEDAI-index and lower complement levels. Moreover, it became evident, that the antibodies correlated with cutaneous manifestations. In the present study, we also analysed for the first time the effect of immunosuppressive therapy on IgE-autoantibody reactivity in several CTD, showing that only IgE-dsDNA antibodies but not the other ANA-specificities were significantly influenced, an observation, that was not unexpected, because this is already known for the IgG-antibodies. Of course, it still has to be confirmed in prospective studies, whether the analysis of IgE-autoantibodies in routine practice can help assess the clinical activity of CTD Nonetheless, the data underscore the importance of IgE autoantibodies in the pathophysiology of CTD justifying the improvement of therapeutic strategies that interact with the production, circulation, or binding of IgE globulins.

## Data Availability

The raw data supporting the conclusions of this article will be made available by the authors, without undue reservation.
