# Supplementary material for: IgE autoantibodies to nuclear antigens in patients with different connective tissue diseases: re-evaluation and novel findings
Source: Front Immunol. 2025 Mar 20;16:1483815. doi: 10.3389/fimmu.2025.1483815 (PMC11965355; doi:10.3389/fimmu.2025.1483815)
Supplement: Supplementary file 1 [file DataSheet1.docx]

Supplementary Material

# Supplementary Table

Table S1a: Clinical parameters in patients with different connective tissue disorders (CTDs) included in the study.

| Clinical parameter | Patients with | | |
| --- | --- | --- | --- |
|  | SLE (n=110) | SS (n=118) | MCTD (n=41) |
| Sex distribution female:male | 7.2:1 | 12.8:1 | 9:1 |
| Age at initial sample collection in years: median (range) | 36  (13-74) | 51  (20-79) | 36  (10-70) |
| Time between first diagnosis and serological analysis in months: median (range) | 45  (-73-355) | 74  (0-270) | 48  (-9-341) |
| Steroids and/or immunsuppressive therapy^*)^: number (%) | 77 (70) | 63 (53) | 31 (76) |
| Frequency of IgG-antibodies to |  |  |  |
| SSA/Ro52 | 54 (49) | 100 (85) | 12 (29) |
| SSA/Ro60 | 60 (55) | 101 (86) | 6 (41) |
| SSB/La | 27 (25) | 49 (42) | 2 (5) |
| Sm/RNP B | 27 (25) | 21 (18) | 13 (32) |
| U1-C RNP | 23 (21) | 19 (16) | 20 (49) |
| RNP68 | 13 (12) | 6 (5) | 26 (63) |
| RNP A | 29 (26) | 18 (15) | 18 (44) |

^*)^ methotrexate, cyclophosphamide, azathioprine, mycophenolate mofetil

Table S1b: Number serum samples from patients with SLE, SS, and MCTD tested by ELISA for IgE -antibodies to different nuclear antigens

| Antigens used | SLE | SS | MCTD |
| --- | --- | --- | --- |
|  | Number sera tested | | |
| dsDNA | 44 | 0 | 0 |
| SSA/Ro52 | 59 | 91 | 27 |
| SSA/Ro60 | 62 | 99 | 26 |
| SSB/La | 35 | 50 | 24 |
| Sm/RNP B | 21 | 25 | 12 |
| U1C-RNP | 25 | 25 | 21 |
| RNP 68 | 26 | 27 | 30 |
| RNP A | 25 | 25 | 21 |

Table S2 Association of IgE- and IgG antibodies to different nuclear antigens in patients with collagen disorders

A) Association of anti-dsDNA-antibodies of the IgG- and IgE type in 44 patients with SLE

| anti-dsDNA | | SLE (n=44) |
| --- | --- | --- |
| IgG | IgE | Number (%) positive |
| + | + | 29 (66) |
| + | - | 15 (34) |
| - | + | 0 |
| - | - | 0 |

B) Association of anti-SSA/Ro52-antibodies of the IgG- and IgE type in patients with SLE and SS

| anti-SSA/Ro52 | | SLE (n= 35) | SS (n= 48) |
| --- | --- | --- | --- |
| IgG | IgE | Number (%) positive | |
| + | + | 17 (49) | 21 (44) |
| + | - | 15 (43) | 25 (52) |
| - | + | 0 | 0 |
| - | - | 3 (9) | 2 (4) |

C) Association of anti-SSA/Ro60-antibodies of the IgG- and IgE type in patients with SLE and SS

| Anti-SSA/Ro68 | | SLE (n= 39) | SS (n= 54) |
| --- | --- | --- | --- |
| IgG | IgE | Number (%) positive | |
| + | + | 13 (33) | 14 (26) |
| + | - | 24 (61) | 36 (67) |
| - | + | 0 | 0 |
| - | - | 2 (5) | 4 (7) |

D) Association of anti-SSB/La-antibodies of the IgG- and IgE type in patients with SLE- and SS

| anti-SSB/La | | SLE (n= 20) | SS (n= 41) |
| --- | --- | --- | --- |
| IgG | IgE | Number (%) positive | |
| + | + | 9 (45) | 29 (71) |
| + | - | 9 (45) | 6 (15) |
| - | + | 0 | 1 (2) |
| - | - | 2 (10) | 5 (12) |

E) Association of Sm/anti-RNP B-antibodies of the IgG- and IgE type in with SLE and MCTD

| Anti-Sm/RNP B | | SLE (n= 14) | MCTD (n= 12) |
| --- | --- | --- | --- |
| IgG | IgE | Number (%) positive | |
| + | + | 10 (71) | 3 (25) |
| + | - | 3 (21) | 6 (50) |
| - | + | 0 | 2 (17) |
| - | - | 1 (7) | 1 (8) |

F) Association of anti-U1-C RNP-antibodies of the IgG- and IgE type in patients with MCTD and SLE

| anti-U1C-RNP | | MCTD (n= 20) | SLE (n= 14) |
| --- | --- | --- | --- |
| IgG | IgE | Number (%) positive | |
| + | + | 11 (55) | 6 (43) |
| + | - | 4 (20) | 1 (7) |
| - | + | 3 (15) | 4 (29) |
| - | - | 2 (10) | 3 (21) |

G) Association of anti-RNP 68-antibodies of the IgG- and IgE type in patients with MCTD and SLE

| anti-RNP68 | | MCTD (n= 29) | SLE (n= 16) |
| --- | --- | --- | --- |
| IgG | IgE | Number (%) positive | |
| + | + | 13 (45) | 4 (25) |
| + | - | 13 (45) | 6 (38) |
| - | + | 0 | 0 |
| - | - | 3 (10) | 6 (38) |

H) Association of anti-RNP A-antibodies of the IgG- and IgE type in patients with MCTD and SLE

| anti-RNP A | | MCTD (n= 19) | SLE (n= 15) |
| --- | --- | --- | --- |
| IgG | IgE | Number (%) positive | |
| + | + | 14 (74) | 12 (80) |
| + | - | 2 (11) | 3 (20) |
| - | + | 3 (16) | 0 |
| - | - | 0 | 1 (7) |

Table S3: Association of anti-dsDNA-,SSA/Ro52, -SSA/Ro60, -SSB/La, -U1C-RNP, -RNP68, -RNPA, and -RNPB(Sm) antibodies of the IgE type with different clinical manifestations and laboratory parameters in 90 patients with SLE

| **Organ manifestations / clinical symptoms** | | auto-antibodies of the IgE type against | | | | | | | |
| --- | --- | --- | --- | --- | --- | --- | --- | --- | --- |
|  |  | dsDNA | SSA/ Ro52 | SSA/Ro60 | SSB/ La | Sm/RNP B | U1C-RNP | RNP 68 | RNP A |
|  |  | number positive / number examined patients (% positive) | | | | | | | |
| Number of organ manifestations | 0-2  >3 | 13 / 24 (54)  14 / 19 (74) | 7 / 16 (44)  10 / 19 (53) | 4 / 19 (21)  8 / 20 (40) | 4 / 9 (44)  4 / 11 (36) | 4 / 7 (57)  6 / 7 (86) | 4 / 7 (57)  6 / 7 (86) | 4 / 10 (40)  0 / 6 (0) | 6 / 9 (67)  6 / 7 (86) |
| SLEDAI | 0  1-3  >=4 | 3 / 4 (75)  6 / 13 (46)  17/25 (68) | **1 / 10 (10)**  **7 / 9 (78) ^**)^**  **8 / 15 (53) ^*)^** | 3 / 12 (25)  5 / 10 (50)  5 / 17 (29) | 4 / 5 (80)  2 / 7 (29)  3 / 8 (38) | 1 / 1 (100)  1 / 2 (50)  8 / 11 (73) | 1 / 1 (100)  2 / 3 (67)  7 / 10 (70) | 0 / 1 (0)  2 / 5 (40)  2 / 10 (20) | 1 / 2 (50)  3 / 3 (100)  8 / 11 (73) |
| cutaneous | without  with | 11 / 22 (50)  15 / 21 (68) | 3 / 12 (25)  14 / 23 (61) | 3 / 13 (23)  9 / 26 (35) | 2 / 5 (40)  8 / 15 (53) | 3 / 3 (100)  7 / 11 (67) | 3 / 4 (75)  7 / 10 (70) | 2 / 6 (33)  2 / 10 (20) | 2 / 5 (40)  10 / 11 (91) |
| musculo-skeletal | without  with | 15 / 26 (58)  12 / 17 (71) | 11 / 17(65)  6 / 18 (33) | 6 / 17 (35)  7 / 22 (32) | 3 / 10 (30)  6 / 10 (60) | 5 / 8 (63)  5 / 6 (83) | 5 / 7 (71)  5 / 7 (71) | 2 / 9 (22)  2 / 7 (29) | 6 / 8 (75)  6 / 8 (75) |
| pulmonary | without  with | 26 / 41 (63)  1 / 2 (50) | 17 / 35 (49)  0 / 0 (0) | 13 / 38 (34)  0 / 1 (0) | 9 / 20 (45)  0 / 0 (0) | 9 / 13 (69)  1 / 1 (100) | 9 / 13 (69)  1 / 1 (100) | 4 / 15 (27)  0 / 1 (0) | 11/15 (73)  1 / 1 (100) |
| cardiac / cardio-vascular | without  with | 26 /41 (63)  1 / 2 (50) | 17 / 35 (49)  0 / 0 (0) | 13 / 38 (34)  0 / 1 (0) | 9 / 20 (45)  0 / 0 (0) | 10 /14 (71)  0 / 0 (0) | 10 / 14 (71)  0 / 0 (0) | 4 / 16 (25)  0 / 0 (0) | 12 / 16 (75)  0 / 0 (0) |
| renal | without  with | 17 / 29 (59)  10 / 14 (71)^#)^ | 14 / 27 (52)  3 / 8 (38) | 9 / 31 (29)  4 / 8 (50) | 7 / 15 (47)  2 / 5 (40) | 8 / 12 (67)  2 / 2 (100) | 8 / 11 (73)  2 / 3 (67) | 4 / 14 (29)  0 / 2 (0) | 11 / 14 (79)  1 / 2 (50) |
| gastro-intestinal | without  with | 27 / 43 (63)  0 / 0 (0) | 17 / 34 (50)  0 / 1 (0) | 13 /37 (35)  0 / 2 (0) | 8 / 19 (42)  1 / 1 (100) | 10/14 (71)  0 / 0 (0) | 10/14 (71)  0 / 0 (0) | 4 / 16 (25)  0 / 0 (0) | 12/16 (75)  0 / 0 (0) |
| neuropsychiatric – non-vascular | without  with | 24 / 40 (60)  3 / 3 (100) | 16 / 33 (48)  1 / 2 (50) | 12 / 38 (32)  1 / 1 (100) | 8 / 19 (42)  1 / 1 (100) | 9 / 13 (69)  1 / 1 (100) | 9 / 13 (69)  1 / 1 (100) | 4 / 15 (27)  0 / 1 (0) | 11 / 15 (73)  1 / 1 (100) |
| neuropsychiatric – vascular | without  with | 24 / 39 (62)  3 / 4 (75) | 17 / 35 (49)  0 / 0 (0) | 13 / 39 (33)  0 / 0 (0) | 9 / 20 (45)  0 / 0 (0) | 10 / 14 (71)  0 / 0 (0) | 10 / 14 (71)  0 / 0 (0) | 4 / 16 (25)  0 / 0 (0) | 12 / 16 (75)  0 / 0 (0) |
| vasculopathy | without  with | 26 / 41 (63)  1 / 2 (50) | 15 / 31 (48)  2 / 4 (50) | 11 / 33 (33)  2 / 6 (33) | 8 / 16 (50)  1 / 4 (25) | 8 / 12 (67)  2 / 2 (100) | 8 / 12 (67)  2 / 2 (100) | 4 / 14 (29)  0 / 2 (0) | 10 / 14 (71)  2 / 2 (100) |
| serositis | without  with | 22 / 33 (67)  5 / 10 (50) | 14 / 25 (56)  3 / 10 (30) | 9 / 29 (31)  4 / 10 (40) | 7 / 16 (44)  2 / 4 (50) | 7 / 11 (64)  3 / 3 (100) | 8 / 11 (73)  2 / 3 (67) | 4 / 13 (31)  0 / 3 (0) | **11 / 12 (92)^*)^**  **1 / 4 (25)** |
| Sicca Syndrome | without  with | 26 / 40 (65)  1 / 3 (33) | 13 / 29 (45)  4 / 6 (67) | 11 / 33 (33)  2 / 6 (33) | 7 / 16 (44)  2 / 4 (50) | 9 / 13 (69)  1 / 1 (100) | 9 / 13 (69)  1 / 1 (100) | 4 / 15 (27)  0 / 1 (0) | 11 / 15 (73)  1 / 1 (100) |
| **Other parameters** | |  |  |  |  |  |  |  |  |
| (secondary) anti-phospho-lipid syndrome | without  with | 15 / 27 (56)  12/16 (75) | 15 / 31 (48)  2 / 4 (50) | 12 / 36 (33)  1 / 3 (33) | 9 / 19 (47)  0 / 1 (0) | 9 / 12 (75)  1 / 2 (50) | 8 / 12 (67)  2 / 2 (100) | 4 / 14 (29)  0 / 2 (0) | 10 / 14 (71)  2 / 2 (100) |
| hematological | without  with | 17 / 25 (68)  10/18 (55) | **6 / 20 (30) ^*)^**  **11/15 (73)** | 7 / 22 (32)  6 / 17 (35) | 6 / 12 (50)  3 / 8 (38) | 6 / 7 (86)  4 / 7 (57) | 5 / 7 (71)  5 / 7 (71) | 2 / 8 (25)  2 / 8 (25) | 7 / 9 (78)  5 / 7 (71) |
| CRP | normal  increased | 12 / 19 (63)  12 / 20 (60) | 8 / 19 (42)  6 / 11 (55) | 5 / 22 (23)  5 / 14 (36) | 5 / 14 (36)  2 / 3 (67) | 4 / 7 (57)  3 / 4 (75) | 3 / 6 (50)  6 / 6 (100) | 4 / 9 (44)  0 / 5 (0) | 8 / 10 (80)  3 / 4 (75) |
| C3 complement | normal  decreased | 10 / 18 (56)  12 / 18 (67) | **2 / 12 (17) ^**)^**  **12 / 15 (80)** | 2 / 14 (14)  7 / 20 (35) | 3 / 6 (50)  3 / 8 (38) | 2 / 4 (50)  5 / 7 (71) | 3 / 5 (60)  5 / 6 (83) | 2 / 5 (40)  1 / 6 (17) | 4 / 5 (80)  6 / 7 (86) |
| C4 complement | normal  decreased | 12 / 23 (52)  9 / 12 (75) | **6 / 18 (33) ^*)^**  **8 / 9 (89)** | 4 / 18 (22)  5 / 14 (36) | 4 / 6 (67)  2 / 7 (29) | 5 / 7 (71)  3 / 5 (60) | 6 / 7 (86)  2 / 4 (50) | 2 / 7 (29)  1 / 4 (25) | 6 / 7 (86)  3 / 4 (75) |
| creatinin | normal  increased | 22 / 34 (65)  4 / 7 (57) | 13 / 25 (52)  0 / 2 (0) | 9 / 29 (31)  1 / 3 (33) | 6 / 15 (40)  1 / 1 (100) | 8 / 11 (73)  0 / 0 (0) | 8 / 11 (73)  0 / 0 (0) | 4 / 13 (31)  0 / 0 (0) | 10/13 (77)  0 / 0 (0) |
| proteinuria | without  with | 20 / 34 (59)  7 / 9 (78) | 14 / 30 (47)  3 / 5 (60) | 10 / 32 (31)  3 / 7 (43) | 8 / 16 (50)  1 / 4 (25) | 8 / 12 (67)  2 / 2 (100) | 9 / 13 (69)  1 / 1 (100) | 4 / 15 (27)  0 / 1 (0) | 11/15 (73)  1 / 1 (100) |

^*)^ p<0,05; ^**)^ p<0,01 as compared to patients with the respective clinical manifestation

^#)^ kidney biopsy had been performed in only 7 of the 14 patients with renal manifestation

Significant data are printed in bold

Table S4: Association of anti-SSA/Ro52, -SSA/Ro60 and -SSB/La antibodies of the IgE type with different clinical manifestations and laboratory parameters in 55 patients with Sjogren syndrome

| **Organ manifestations / clinical symptoms** | | auto-antibodies of the IgE type against | | |
| --- | --- | --- | --- | --- |
|  |  | SSA/Ro52 | SSA/Ro60 | SSB/La |
|  |  | number positive / number of examined patients (% positive) | | |
| Number of organ manifestations | 0-2  >3 | 16 / 38 (42)  5 / 14 (36) | 12 / 39 (31)  3 / 15 (20) | 22 / 29 (76)  7 / 12 (58) |
| Sicca Syndrome | without  with | 3 / 7 (43)  18 / 45 (40) | 1 / 7 (14)  12 / 47 (26) | 3 / 4 (75)  20 / 37 (54) |
| cutaneous | without  with | 19 / 41 (46)  2 / 11 (18) | 12 / 42 (29)  2 / 12 (17) | 27 / 34 (79)  3 / 7 (43) |
| musculoskeletal | without  with | 13 / 35 (37)  8 / 17 (47) | 9 / 35 (26)  5 / 19 (26) | 17 / 24 (71)  13 / 17 (76) |
| pulmonary | without  with | 21 / 48 (44)  0 / 4 (0) | 13 / 50 (26)  1 / 4 (25) | 27 / 37 (73)  3 / 4 (75) |
| cardiac | without  with | 21 / 52 (40)  0 / 0 (0) | 14 / 54 (26)  0 / 0 (0) | 30 / 41 (73)  0 / 0 (0) |
| renal | without  with | 20 / 50 (40)  1 / 2 (50) | 14 / 52 (27)  0 / 2 (0) | 28 / 39 (72)  2 / 2 (100) |
| gastrointestinal | without  with | 21 / 50 (42)  0 / 2 (0) | 14 / 52 (27)  0 / 2 (0) | 29 / 39 (74)  1 / 2 (50) |
| neuropsychiatric | without  with | 18 / 46 (39)  3 / 6 (50) | 12 / 48 (25)  2 / 6 (33) | 26 / 35 (74)  4 / 6 (67) |
| vasculopathy | without  with | 17 / 46 (37)  4 / 6 (67) | 12 / 48 (25)  2 / 6 (33) | 25 / 35 (71)  5 / 6 (83) |
| serositis | without  with | 20 / 50 (40)  1 / 2 (50) | 13 / 52 (25)  1 / 2 (50) | 30 / 41 (73)  0 / 2 (0) |
| lymphadenopathy | without  with | 19 / 48 (40)  2 / 4 (50) | 12 / 50 (24)  2 / 4 (50) | 27 / 37 (73)  3 / 4 (75) |
| **Other parameters** | |  |  |  |
| thyroidal | without  with | 21 / 51 (41)  0 / 1 (0) | 14 / 53 (26)  0 / 1 (0) | 30 / 40 (75)  0 / 1 (0) |
| haematological | without  with | 16 / 43 (37)  5 / 9 (56) | 11 / 45 (24)  3 / 9 (33) | 25 / 36 (69)  5 / 5 (100) |
| (secondary) anti-phospholipid syndrome | without  with | 20 / 50 (40)  1 / 2 (50) | 13 / 52 (25)  1 / 2 (50) | 30 / 40 (75)  0 / 1 (0) |
| CRP | normal  increased | 13 / 37 (35)  6 / 13 (46) | 10 / 38 (26)  3 / 13 (23) | 21 / 28 (75)  7 / 10 (70) |
| C3 complement | normal  decreased | 11 / 31 (35)  1 / 3 (33) | 8 / 31 (26)  0 / 3 (0) | 15 / 22 (68)  2 / 3 (67) |
| C4 complement | normal  decreased | 12 / 33 (36)  1 / 2 (50) | 8 / 33 (24)  1 / 2 (50) | 16 / 24 (67)  2 / 2 (100) |
| Beta2-microglobulin | normal  increased | 0 / 1 (0)  0 / 2 (0) | 0 / 1 (0)  1 / 2 (50) | 1 / 1 (100)  2 / 2 (100) |

Table S5: Association of anti-Sm/RNP B-, -U1C-RNP-, -RNP 68-, and -RNP A antibodies of the IgE type with different clinical manifestations and laboratory parameters in 38 patients with mixed connective tissue disease

| **Organ manifestations / clinical symptoms** | | auto-antibodies of the IgE type against | | | |
| --- | --- | --- | --- | --- | --- |
|  |  | Sm/RNP B | U1C-RNP | RNP 68 | RNP A |
|  |  | number positive / number of examined patients (% positive) | | | |
| Number of organ manifestations | 0-2  >3 | 1 / 3 (33)  4 / 9 (44) | 3 / 5 (60)  11 / 15 (73) | 3 / 11 (27)  10 / 18 (56) | 4 / 5 (80)  13 / 14 (93) |
| cutaneous | without  with | 1 / 3 (33)  4 / 9 (44) | 5 / 7 (71)  9 / 13 (69) | 5 / 12 (42)  8 / 17 (47) | 8 / 8 (100)  9 / 11 (82) |
| Musculo-skeletal | without  with | 1 / 4 (25)  4 / 8 (50) | 1 / 4 (25)  13 / 16 (81) | 3 / 9 (33)  10 / 20 (50) | 4 / 5 (80)  13 / 14 (93) |
| pulmonary | without  with | **2 / 9 (22)^**)^**  **3 / 3 (100)** | 8 / 14 (57)  6 / 6 (100) | 8 / 21 (38)  5 / 8 (63) | 10 / 12 (83)  7 / 7 (100) |
| cardiac | without  with | 4 / 11 (36)  1 / 1 (100) | 13 / 19 (68)  1 / 1 (100) | 12 / 28 (43)  1 / 1 (100) | 16 / 18 (89)  1 / 1 (100) |
| renal | without  with | 3 / 9 (33)  2 / 3 (67) | 11 / 16 (69)  3 / 4 (75) | 11 / 26 (42)  2 / 3 (67) | 12 / 14 (86)  5 / 5 (100) |
| Gastrointestinal^*)^ | without  with | 4 / 11 (36)  1 / 1 (100) | 14 / 19 (74)  0 / 1 (0) | 12 / 27 (44)  1 / 2 (50) | 16 / 18 (89)  1 / 1 (100) |
| neuro-psychiatric | without  with | 5 / 12 (42)  0 / 0 (0) | 14 / 20 (70)  0 / 0 (0) | 13 / 28 (46)  0 / 1 (0) | 17 / 19 (89)  0 / 0 (0) |
| vasculopathy | without  with | 1 / 1 (100)  4 / 11 (36) | 2 / 2 (100)  12 / 18 (67) | 1 / 4 (25)  12 / 25 (48) | 2 / 3 (67)  15 / 16 (94) |
| serositis | without  with | 4 / 10 (40)  1 / 2 (50) | 11 / 17 (65)  3 / 3 (100) | 12 / 26 (46)  1 / 3 (33) | 15 / 17 (88)  2 / 2 (100) |
| Sicca Syndrome | without  with | 3 / 10 (30)  2 / 2 (100) | 13 / 18 (72)  1 / 2 (50) | 12 / 26 (46)  1 / 3 (33) | 14 / 16 (88)  3 / 3 (100) |
| lymphadeno-pathy | without  with | 4 / 10 (40)  1 / 2 (50) | 13 / 18 (72)  1 / 2 (50) | 13 / 26 (50)  0 / 3 (0) | 15 / 17 (88)  2 / 2 (100) |
| **Other parameters** | |  |  |  |  |
| hepatic | without  with | 5 / 12 (42)  0 / 0 (0) | 14 / 20 (70)  0 / 0 (0) | 13 / 29 (45)  0 / 0 (0) | 17 / 19 (89)  0 / 0 (0) |
| haematological | without  with | 3 / 7 (43)  2 / 5 (40) | 11 / 14 (79)  3 / 6 (50) | 7 / 20 (35)  6 / 9 (67) | 10 / 11 (91)  7 / 8 (88) |
| CRP | normal  increased | 2 / 5 (40)  3 / 7 (43) | 6 / 10 (60)  8 / 10 (80) | 4 / 14 (29)  8 / 14 (57) | 5 / 6 (83)  11 / 12 (92) |
| C3 complement | Normal  decreased | 1 / 3 (33)  2 / 6 (33) | 8 / 10 (80)  4 / 6 (67) | 3 / 14 (21)  4 / 6 (67) | 7 / 8 (88)  4 / 5 (80) |
| C4 complement | normal  decreased | 2 / 5 (40)  1 / 4 (25) | 8 / 11 (73)  4 / 5 (80) | 4 / 15 (27)  3 / 5 (60) | 8 / 9 (89)  3 / 4 (75) |

*) including esophageal dysmotility, dyspepsia, esophageal and gastric ulcerations,pseudo-obstruction, bowel ischemia/perforation or enteritis

^**)^ p<0,05 as compared to patients with the respective clinical manifestation

Significant data are printed in bold

# Supplementary Figures

#

**Figure S1**: Correlation between IgG- and IgE-reactivity for antibodies to dsDNA (A), SSA/Ro52 (B), SSA/Ro60 (C), SSB/La (D), Sm/RNP B (E), U1-C RNP (F), RNP 68 (G), and RNP A (H) in all samples which had been tested for the respective antibodies independently from the underlying diagnosis. r=correlation coefficient; OD=optical density; p=significance level; s=slope
